# Usage, Acceptability, and Effectiveness of an Activity Tracker in a Randomized Trial of a Workplace Sitting Intervention: Mixed-Methods Evaluation

**DOI:** 10.2196/ijmr.9001

**Published:** 2018-03-02

**Authors:** Charlotte L Brakenridge, Genevieve N Healy, Elisabeth AH Winkler, Brianna S Fjeldsoe

**Affiliations:** ^1^ School of Public Health The University of Queensland Brisbane Australia; ^2^ Baker Heart and Diabetes Institute Melbourne Australia; ^3^ School of Physiotherapy and Exercise Science Curtin University Perth Australia

**Keywords:** wearable electronic devices, fitness trackers, sedentary lifestyle, exercise, workplace, adult

## Abstract

**Background:**

Wearable activity trackers are now a common feature of workplace wellness programs; however, their ability to impact sitting time (the behavior in which most of the desk-based workday is spent) is relatively unknown. This study evaluated the LUMOback, an activity tracker that targets sitting time, as part of a cluster-randomized workplace sitting intervention in desk-based office workers.

**Objective:**

Study objectives were to explore: (1) office workers’ self-directed LUMOback use, (2) individual-level characteristics associated with LUMOback use, (3) the impact of LUMOback use on activity and sitting behaviors, and (4) office workers’ perceived LUMOback acceptability.

**Methods:**

Exploratory analyses were conducted within the activity tracker intervention group (n=66) of a 2-arm cluster-randomized trial (n=153) with follow-up at 3 and 12 months. The intervention, delivered from within the workplace, consisted of organizational support strategies (eg, manager support, emails) to stand up, sit less, and move more, plus the provision of a LUMOback activity tracker. The LUMOback, worn belted around the waist, provides real-time sitting feedback through a mobile app. LUMOback usage data (n=62), Web-based questionnaires (n=33), activPAL-assessed sitting, prolonged (≥30 min bouts) and nonprolonged (<30 min bouts) sitting, standing and stepping time (7-day, 24 h/day protocol; n=40), and telephone interviews (n=27) were used to evaluate study aims. LUMOback usage data were downloaded and described. Associations between user characteristics and LUMOback usage (in the first 3 months) were analyzed using zero-inflated negative binomial models. Associations between LUMOback usage and 3-month activity outcomes were analyzed using mixed models, correcting for cluster. LUMOback acceptability was explored using 3-month questionnaire data and thematic analysis of telephone interviews (conducted 6 to 10 months post intervention commencement).

**Results:**

Tracker uptake was modest (43/61, 70%), and among users, usage over the first 3 months was low (1-48 days, median 8). Usage was greatest among team leaders and those with low self-perceived scores for job control and supervisor relationships. Greater tracker use (≥5 days vs <5 days) was significantly associated only with changes in prolonged unbroken sitting (−50.7 min/16 h; 95% CI −94.0 to −7.3; *P*=.02) during all waking hours, and changes in nonprolonged sitting (+32.5 min/10 h; 95% CI 5.0 to 59.9; *P*=.02) during work hours. Participants found the LUMOback easy to use but only somewhat comfortable. Qualitatively, participants valued the real-time app feedback. Nonuptake was attributed to being busy and setup issues. Low usage was attributed to discomfort wearing the LUMOback.

**Conclusions:**

The LUMOback—although able to reduce prolonged sitting time—was only used to a limited extent, and its low usage may provide a partial explanation for the limited behavior changes that occurred. Discomfort limited the feasibility of the LUMOback for ongoing use. Such findings yield insight into how to improve upon implementing activity trackers in workplace settings.

## Introduction

Desk-based workers engage in high levels of sedentary time [1,2] (ie, waking behaviors spent in a sitting, reclining, or lying posture with low energy expenditure [3]), putting themselves at risk of musculoskeletal [4] and cardio-metabolic issues [5]. Much of this sitting time is in prolonged, unbroken bouts [1], which have additional cardio-metabolic risks compared with sitting with regular breaks [5]. Furthermore, desk-based workers spend only a small percentage of their workday stepping (<10% of work time) [2] and engage in minimal amounts of moderate to vigorous activity both in and outside of work hours [6]. Given that sedentary work is increasing [7] and workers spend the majority of their day at work [8], there is a need to design and evaluate interventions that target the desk-based workplace.

In recent years, consumer-based activity trackers have become a popular component of workplace wellness programs to increase workers’ physical activity [9], and it is expected that by 2021, 171.9 million activity trackers will be in workplaces worldwide [10]. Most trackers offer features known to be important for behavior change including self-monitoring, real-time feedback and prompting, guided goal setting, and rich and tailored feedback data [11]. Their popular use in workplace wellness programs indicates that they may be a feasible and acceptable intervention strategy for organizations to disseminate [9], and there is emerging evidence to suggest that consumer-based activity trackers can improve physical activity outcomes in desk-based workers [12,13].

Despite these findings, investigation concerning how desk-based workers use consumer-based trackers, which worker characteristics are associated with use, and whether workers find trackers acceptable and appealing has been scarce. The limited evidence suggests that although initial engagement is high [12,13], use may drop off in the long term [13,14]. Tracker users have been shown to fall into distinct groups, with high use seen among young and fit adults, as well as older adults with a desire to improve their health [15]. Acceptability may depend on user motivation and ease of use of the tracker [14]. Furthermore, it is also unknown whether use of a consumer-based activity tracker that targets and provides real-time *sitting* feedback can result in reductions in desk-based workers’ sitting time. Strategies that target increases in activity may not lead to reductions in sitting time [16], and therefore, trackers that specifically target sitting time need to be evaluated.

To address these gaps, this study explored the usage and acceptability of an activity tracker (the LUMOback) that provided real-time feedback on sitting among desk-based office workers participating in a cluster-randomized workplace trial, targeting reductions in prolonged sitting time [17,18]. The LUMOback tracker was chosen over other activity trackers because it is one of the few commercially available trackers that specifically measures and provides real-time sitting feedback [19]. The aim of this study was to explore the following: (1) participants’ LUMOback usage (ie, device wear); (2) which personal, health, job, and activity characteristics were associated with greater LUMOback usage; (3) the relationship between LUMOback usage and changes in sitting and activity outcomes both at work and across all waking hours; and (4) participants’ acceptability and perceptions of the LUMOback.

## Methods

### Participants and Design

Details of the study [17] and the effectiveness outcomes [18] have been reported previously (trial registration: ACTRN12614000252617). Briefly, participants were desk-based office employees of a large international property and infrastructure group. Workers were recruited in teams from 2 locations (A and B). Teams were cluster-randomized to receive either organizational support strategies alone (Group ORG; 9 manager-led teams; 87 participants) or organizational support strategies plus the provision of a LUMOback tracker (Group ORG+Tracker; 9 manager-led teams; 66 participants). The intervention was ongoing; data collection occurred at baseline and 3 and 12 months post intervention commencement. Only the Group ORG+Tracker is examined in this study. The organizational support component, designed to be workplace driven, was delivered by a workplace champion (the organization’s Head of Workplace Wellbeing) and consisted of an information booklet; emails with tips to stand up, sit less, and move more; and involvement of senior executives to support the study. Participants in both groups also received individualized feedback concerning their sitting time and physical activity as recorded by the activPAL after each assessment. The study was approved by the University of Queensland Behavioural and Social Sciences Ethical Review Committee (approval number: 2014000089). All participants provided written informed consent, which covered the 3-month evaluation, with reconsent required to participate in the 12-month evaluation [17].

### The LUMOback Tracker

The activity tracker provided to participants was the LUMOback activity tracker (LUMO Bodytech, Mountain View, CA, USA). The LUMOback is worn as a belt around the lower back (over or under clothing) and provides real-time feedback (through a mobile app) on sitting, standing, sit-to-stand transitions, walking, running, step count, posture, and sleep. Users can set posture alerts that vibrate the belt when the user is in a poor lumbar posture (identified by pelvic tilt angle) and set sitting time push notifications to their phone when sitting for periods of 15 min, 30 min, 45 min, 1 hour, or 2 hours (user-defined). The LUMOback provides valid and reliable measures of sitting time and number of steps [20,21].

LUMOback trackers, along with a 4-page instruction booklet, were distributed by the workplace champion in study week 1. Participants set up their LUMObacks in their own time but had the contact details of the project coordinator and the workplace champion for any questions. LUMOback setup required calibration to the user’s sitting and standing posture, and this was guided through the app and booklet instructions. The LUMOback was originally designed as a posture rather than as a sitting device [19]. As such, the default settings of the tracker were to have vibrating alerts (based on posture) on and sitting notifications off. To be consistent with evaluating the use of the tracker as it would be used outside of the research context, these settings were not preadjusted by project staff; however, the booklet instructions indicated that both these settings could be changed by the participant if the participant wished. LUMOback data were downloaded by project staff and monitored for usage during the study period. No specific instruction was given to participants on how much to wear the LUMOback; however, participants who did not have any LUMOback usage data were followed up by the project coordinator (via email or phone) or by the workplace champion (in person) to troubleshoot issues about setup during weeks 3 to 10. Participants were permitted to keep the LUMOback after the study ended.

### Data Collection

Usage data were collected from LUMO Bodytech every 2 weeks during the initial 3 months and periodically thereafter. Participants completed Web-based questionnaires (via LimeService, used to address aims 2 and 4) at baseline, 3 months, and 12 months. The questionnaires collected data on sociodemographics (baseline), health and job characteristics (baseline, 3 months, and 12 months), LUMOback acceptability (3 months), and usage of behavior change techniques (eg, goal setting and prompting) in the LUMOback app (3 and 12 months). Height and weight measurements were collected objectively in person at the baseline assessment or via self-report (used in aim 2). Sitting and activity data (used to address aims 2 and 3) were collected at baseline, 3 months, and 12 months via the activPAL3 activity monitor (PAL Technologies Ltd., Glasgow, Scotland, UK). Telephone interviews, conducted 6 to 10 months after intervention commencement, were used to collect qualitative data regarding the LUMOback’s acceptability to participants (used to address aim 4). Because so few participants reconsented for the 12-month assessment, aims 2 and 3 consider only usage and outcomes over the first 3 months.

### Measures

#### Personal, Health, and Job Characteristics

Key participant characteristics, as measured at baseline, were age, gender, highest level of education completed, job category (senior or team leader, managerial staff, other general staff), use of other activity-promoting apps and wearable devices (yes or no), confidence using a smartphone (1-5 scale, least to most confidence), desired sitting at work (≥50%, <50% of work time) [22], knowledge of the health impacts of sitting (1-5 scale, least to most knowledge) [22], musculoskeletal symptoms (Nordic Musculoskeletal Questionnaire [23], categorized as problems over the last month [yes or no] in the upper body, back, and lower extremities), and physical and mental health-related quality of life (Short Form-12 version 1 [24], 0-100 scale) and several other health- and job-related scores, all measured on a 1-10 scale. Specifically, these were stress [25], job control [25], work satisfaction [25], supervisor relations [25] (all from the Health and Work Questionnaire), and job performance (self-rated job performance scale [26]). For all of the health- and job-related scores, apart from stress, higher values are desirable. Height and weight measurements were used to calculate body mass index at baseline.

#### Activity

Sitting and activity data were collected via the activPAL3 monitor. At each assessment, participants were asked to wear the monitor for 7 days, 24 hours per day. Further information on monitor processing has been provided in previous trial publications [17,18]. Activity outcomes considered here are time per 10 hours at work and time per 16 hours awake spent engaged in sitting, prolonged sitting (≥30 min continuously), nonprolonged sitting (<30 min continuously), standing, and stepping. To take into account variations in work times between assessments, and between individuals, work hour outcomes were standardized to a 10-hour workday by multiplying by 10 hours and dividing by work wear time. Similarly, overall waking hour outcomes were standardized to a 16-hour day by multiplying by 16 hours and dividing by waking wear time. Work and waking times were collected by self-report.

#### LUMOback Usage

For participants who set up the LUMOback, data on LUMOback usage were downloaded as comma-separated value files from LUMO Bodytech. Each file contains date and time-stamped summaries per 5 min time interval on activity and posture in various metrics, and an indicator of whether the LUMOback was not worn or charging. Daily usage (hours/day) was estimated by calculating the time elapsed between the first and last 5-min time window each day during which the LUMOback recorded an activity other than lying down, and then subtracting from this usage time any probable device nonwear time (or sleep time) occurring during this period. Only days with ≥1 hour of usage were counted as days of usage. Participants with at least 1 day of usage were classed as LUMOback “users”; those with no days of usage, or who self-reported never using the LUMOback, were classed as “nonusers.” Others who reported using the LUMOback but lacked usage data were classed as having an “unknown” degree of usage.

LUMOback usage over the initial 3 months of intervention was considered: continuously (days), as a binary classification (≥5 days or <5 days), and as a 4-category classification (nonuser, limited user, infrequent user, or frequent user). The thresholds used for the 4-category classification were 0, 1-4, 5-15, and ≥16 days, respectively, over 3 months. Long-term (12-month) usage was also assessed for the subset of participants who had opted for the 12-month assessment. LUMOback usage during the activity monitor assessment (yes or no) was also considered to provide some insight as to whether the timing of LUMOback wear (not just how much it was worn) was important for activity changes. Usage/nonusage during the activity monitor assessment was determined by the presence/absence of ≥1 day with ≥1 hour of usage over this time frame, or by self-report when LUMOback data were not available.

#### LUMOback Acceptability

All questionnaire items assessing LUMOback acceptability were created for the trial. Acceptability questionnaire items were closed response (1, not at all; 2, somewhat; 3, not sure; 4, comfortable/easy; and 5, extremely comfortable/easy) and assessed for the following: comfort of the LUMOback, the ease of setup of the LUMOback and app, ease of navigation and use of the app, and ease of LUMOback calibration to the user’s posture. Participants’ perceived usefulness of the LUMOback overall and of specific features such as the graphs, sitting notifications, and vibrating alerts were also assessed in closed-response items: 1, not at all; 2, somewhat; 3, not sure; 4, useful; 5, extremely useful; and, when relevant, 6, did not notice/use. Frequency of use of the behavior change features was also assessed (eg, “When wearing your LUMOback, how often did you have it set to vibrate?”), with response options from 1 (never) to 6 (all the time). The full list of questions is provided in Multimedia Appendix 1 of the study protocol [17].

All participants who had not withdrawn after the 3-month assessment were eligible to take part in the telephone interviews. To ensure representation across teams, it was planned that at least 2 participants per team would be interviewed, plus the team managers. Interviews continued until the data were saturated and at least 2 members per work team were interviewed, unless there were more than 5 unsuccessful attempts to interview. The lead author (CLB, in location B) conducted and recorded (using Audacity) one-on-one telephone interviews with participants (predominantly in location A), ranging in duration from 9 to 28 min (mean duration 14 min). These semi-structured interviews covered topics of general thoughts on the LUMOback, participant likes and dislikes, barriers to usage, use of the sitting notification, influence of work team on LUMOback use, and general activity tracker preferences.

### Sample Size

The sample size was determined based on the needs of the cluster-randomized trial [17]. This study was exploratory and not powered on study aims. Minimum differences of interest (MDI) in this study were 30 min of sitting and standing and 15 min of stepping between LUMOback usage categories (≈per 10 days of usage), and 2 days of LUMOback usage between groups (categorical variables), per amount of activity equivalent to the above, or per 1 standard deviation of other continuous variables.

### Analysis

Statistical analyses were performed in STATA version ≥13 (StataCorp, College Station, TX, USA). Significance was set at *P*<.05 2-tailed. Analyses were of all evaluable cases with missing or “unknown” data excluded. Mean and standard deviation were reported for continuous variables, and percentage was reported for categorical variables.

To address aim 1, usage was described. In the case of non-normally distributed variables, the median and the 25th and 75th percentiles were reported in addition to the mean and SD. To address aim 2, associations of characteristics with LUMOback use (adjusting for age, sex, and location) were statistically tested using zero-inflated negative binomial models (which accounted for the excessive zeros, as indicated by the Vuong test). Results are reported as contrasts of marginal mean outcomes. To address aim 3, associations of LUMOback usage with activity outcomes were examined using linear mixed models, adjusting for baseline value of the outcome, age, and sex and correcting for team cluster. Models were checked for nonlinearity, non-normality of residuals, multicollinearity, heteroscedasticity, and influential cases. Outcomes were transformed when this improved models, with the results from these models presented back-transformed to regular units. Results are presented with and without influential cases, if these cases were found to change the interpretation of the results.

To address aim 4, acceptability of the LUMOback was described quantitatively, based on the questionnaire items, and qualitatively, based on thematic analysis of interview data. Qualitative interviews were transcribed verbatim using F4 software (audiotranskription, Marburg, Germany). Participant idiosyncrasies (eg, “um”) were removed after transcription. Author CLB coded initial themes (using NVivo version 10 and Microsoft Office Word). These were then compared and contrasted with a second researcher and discussed and decided upon. Themes were not preidentified; however, some of the questions in the semi-structured interview had a narrow focus that heavily contributed to some of the themes. Themes were then compared and contrasted with the questionnaire data when these were available, with findings from both sources presented together.

## Results

### Participant Characteristics

Baseline characteristics of participants in the ORG+Tracker group (overall and by usage) are presented in Multimedia Appendix 1. Participants (35 female and 31 male) on average were aged 37.6 (SD 7.8) years and engaged in high levels of sitting time during work hours (453.0 [SD 55.9] min/10 h, ie, 76% of work hours). The majority were university educated (54/63, 86%); either in managerial (38/66, 58%), team or senior leader (8/66, 12%), or general staff (20/66, 30%) positions; and confident to extremely confident using a smartphone (52/54, 96%).

### Aim 1: LUMOback Uptake and Usage

#### Uptake

The LUMOback was provided to 61 participants. Overall, 5 participants did not receive the LUMOback because of either ineligibility (incompatible phone, not ambulatory, left organization; n=3) or refusal (n=1), or for an unknown reason (n=1; see Multimedia Appendix 2 for flow diagram). Uptake was modest overall (43/66, 65%) and within those provided a LUMOback (43/61, 70%). Nonusers’ (n=18) reported reasons for nonuse were as follows: being too busy to set up (6/18, 33%), personal disruptions during the study period (4/18, 22%), having technical difficulties setting up (3/18, 17%), pregnancy (1/18, 6%), and unknown (4/18, 22%). An additional “user” was later classed as a nonuser as they only had a very small and invalid amount of usage data (<1 hour), bringing the total number of nonusers to 19. Nonusers of the LUMOback were less likely to provide 3-month follow-up activity data than LUMOback users (see Multimedia Appendix 2).

#### Short-Term Usage

Out of the remaining 42 users, 4 participants claimed to have used the LUMOback, but usage data could not be obtained from LUMO Bodytech. Among those whose usage data could be collected (n=38), the LUMOback was used over the first 3 months for 1 to 48 of a possible 91 days, with a mean of 11.7 (SD 11.8) days of use and median (25th, 75th percentile) of 8 (3, 15) days. As usage can be intermittent, the time from first to last recorded usage within the first 3 months was slightly longer than the days of usage, ranging from 1 to 78 days, with a mean of 24.5 (SD 21.3) days and median (25th, 75th percentile) of 17.5 (7, 38) days. Duration of usage on wear days averaged 9.8 (SD 3.7) h per day.

**Figure 1:**
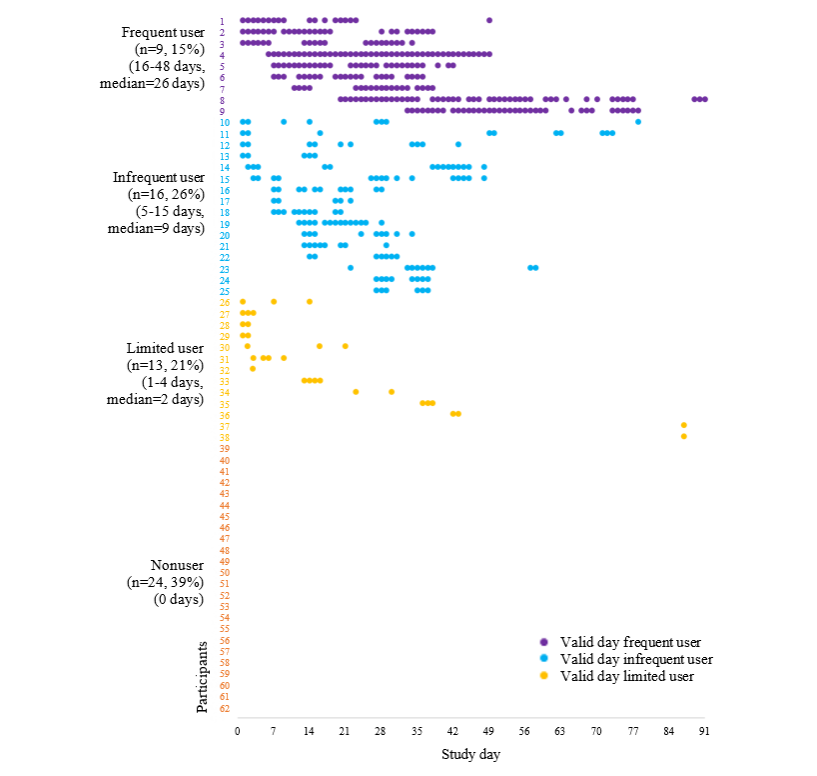
LUMOback use by study day over the first 3 months (91 days), ordered by first study day of use and total length of use within each usage category. Percentages do not add to 100% due to rounding.

Figure 1 demonstrates the diverse patterns of usage during the first 3 months of the study by usage group. Only 9 participants (15%) were classed as frequent users, whereas the remaining participants were either infrequent users (16/62, 26%), limited users (13/62, 21%), or nonusers (24/62, 39%). Duration of usage on wear days (h/day) appeared to be longest in frequent users (mean 12.8, SD 1.6), followed by infrequent users (mean 10.0, SD 3.2), and then by limited users (mean 7.4, SD 3.8).

#### Long-Term Usage

LUMOback usage after the 3-month assessment was evaluated in the 21 LUMOback users who reconsented to 12-month follow-up (reasons for nonconsent were not related to LUMOback usage; see Multimedia Appendix 2). Only 1 consenting participant used their LUMOback after the 3-month assessment, for 5 consecutive additional days in total, with the last day of use being day 185 (ie, approximately 6 months after intervention commencement).

### Aim 2: Participant Characteristics Associated With Short-Term LUMOback Usage

**Figure 2:**
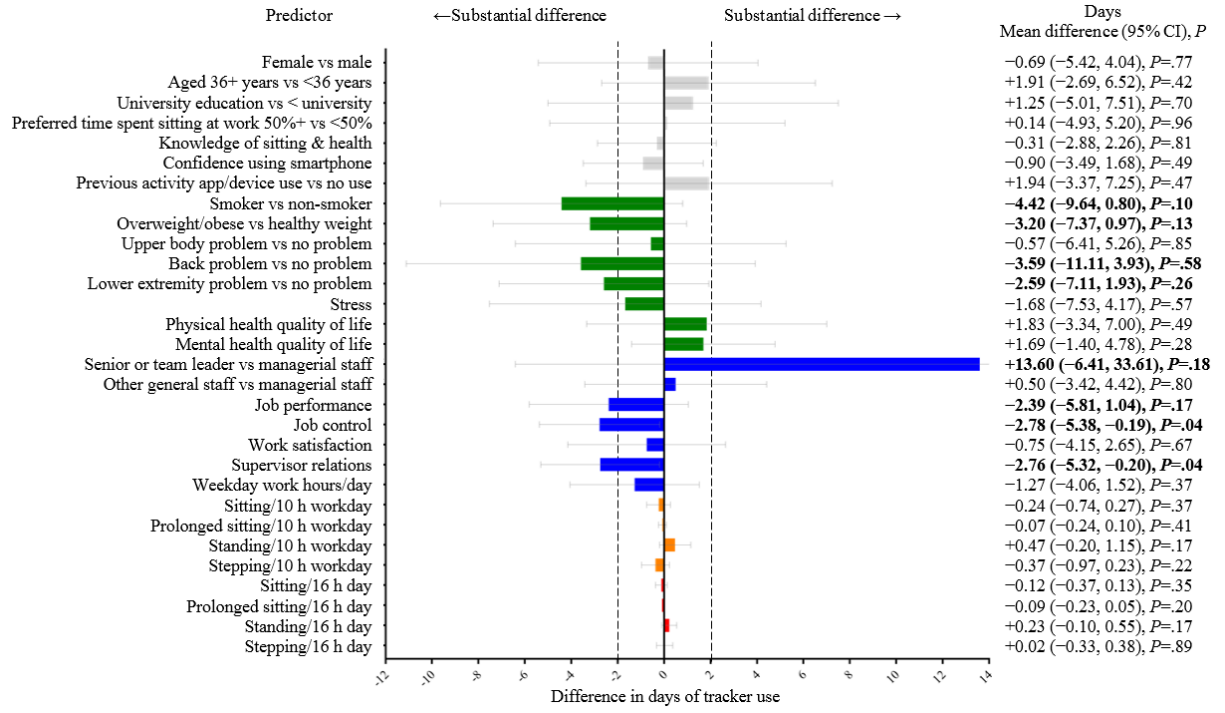
Predictors of number of days of LUMOback usage over the first 3 months (91 days) of the intervention. (Gray indicates personal characteristics, green indicates health characteristics, blue indicates job characteristics, orange indicates work activity characteristics, and red indicates overall activity characteristics. Boldface refers to difference ≥ the minimum difference of interest. Marginal mean [95% CI] days of LUMOback use [zero inflated negative binomial models], adjusted for sex, age, and location contrasted between groups [categorical variables] or mean versus “mean + dose” [continuous variables]. Dose was 1 SD for most continuous variables, 6 min for sitting and standing, and 3 min for stepping, per day or workday. The CI for senior or team leader continues off figure.).

Figure 2 shows the number of days of LUMOback use compared across personal, health, job, and activity characteristics. Job control and supervisor relations were the only statistically significant predictors of usage. For both variables, higher perceived scores were associated with fewer days of LUMOback usage (~3 days). Some of the nonsignificant differences were substantial (≥the MDI). Specifically, being a senior or team leader rather than managerial staff was meaningfully associated with more days (~14 days) of LUMOback usage, whereas having higher perceived job performance, being a smoker, being overweight or obese, and having back or lower extremity musculoskeletal problems were meaningfully associated with fewer days (~2 to 4 days) of LUMOback usage. Other differences were small (<the MDI).

### Aim 3: Impact of Short-Term LUMOback Usage on Behavior Change

LUMOback usage for at least 5 days during the intervention period was significantly associated with more nonprolonged sitting time during work hours (+32.5 min/10 h workday, 95% CI 5.0 to 59.9, *P*=.02). An examination of Cook’s distance (*D*) identified 2 influential cases (Cook’s *D* ≥.14), and after removal of these cases, the association with nonprolonged sitting time was no longer statistically significant, although still tended in the same direction (+19.6 min/10 h, 95% CI −3.6 to 42.7, *P*=.10). Increases in nonprolonged sitting time were coupled with less prolonged sitting time (−38.1 min/10 h, 95% CI −80.1 to 3.9, *P*=.08), and only very small differences in sitting, standing, and stepping time changes during work hours (see Table 1).

During waking hours, those using the LUMOback for at least 5 days reduced their prolonged sitting time by significantly more than their lower usage counterparts (−50.7 min/16 h awake, 95% CI −94.0 to −7.3, *P*=.02). This comparatively greater shift in waking hours away from prolonged sitting time was coupled with a shift toward more nonprolonged sitting time (+25.4 min/16 h, 95% CI −5.7 to 56.6, *P*=.11), which became significant after the removal of 1 influential case (Cook’s *D*=.25; +31.9 min/16 h, 95% CI 3.3 to 60.4, *P*=.03). In addition, there remained some small differences in sitting (−29.4 min/16 h, 95% CI −67.2 to 8.4, *P*=.13), standing (+21.1 min/16 h, 95% CI −10.7 to 52.9, *P*=.19), and stepping (+4.6 min/16 h, 95% CI −9.2 to 18.3, *P*=.51) changes, all favoring those who used the LUMOback for at least 5 days.

**Table 1:** Associations of LUMOback usage with 3-month activity changes during the intervention.

Outcome		Used during the intervention for ≥5 days, yes vs no (adjusted mean difference, 95% CI)^a^	*P* value	Usage during activity assessment, yes vs no (adjusted mean difference, 95% CI)^a^	*P* value
**Work hours, min/10 h workday^b^**					
	Sitting	−1.9 (−22.0 to 18.1)^c^	.85	−7.5 (−27.8 to 12.9)^c^	.47
	Prolonged sitting	−38.1 (−80.1 to 3.9)	.08	−28.3 (−74.0 to 17.4)	.23
	Nonprolonged sitting	*32.5 (5.0 to 59.9)*	*.02*	24.7 (−8.0 to 57.4)	.14
	Standing	0.7 (−17.3 to 18.7)	.94	−1.5 (−18.8 to 15.7)	.86
	Stepping	2.6 (−7.5 to 12.8)	.61	5.3 (−4.4 to 15.0)	.29
**All waking hours, min/16 h day^b^**					
	Sitting	−29.4 (−67.2 to 8.4)	.13	−2.0 (−40.0 to 36.0)	.92
	Prolonged sitting	− *50.7 (−94.0 to −7.3)*	*.02*	−35.1 (−79.4 to 9.2)	.12
	Nonprolonged sitting	25.4 (−5.7 to 56.6)	.11	*36.5 (7.5 to 65.6)*	*.01*
	Standing	21.1 (−10.7 to 52.9)	.19	4.2 (−27.1 to 35.5)	.79
	Stepping	4.6 (−9.2 to 18.3)	.51	−1.0 (−14.2 to 12.2)	.89

^a^Table shows adjusted mean difference and 95% CI from linear mixed models, adjusting for baseline value of the outcome, age, and sex, and correcting for cluster. Italics indicate statistically significant association (set at *P*<.05 2-tailed).

^b^Analyzes are of evaluable cases among the 66 ORG+Tracker participants, excluding those with missing activity outcomes at work (n=30), during waking hours (n=26), or with unknown usage during the intervention (n=3). Work hours analyses include 18 users and 15 nonusers during the intervention and 21 users and 15 nonusers during the assessment while waking hours analyses include 19 users and 18 nonusers during the intervention and 22 users and 18 nonusers during the assessment.

^c^Sitting time during work hours was modeled as natural log of (600 minutes minus sitting minutes); results are back-transformed to original units for presentation in the table.

Examining usage of the LUMOback specifically over the time frame that activity was being measured revealed that concurrent usage was significantly associated with more nonprolonged sitting time during waking hours (+36.5 min/16 h, 95% CI 7.5 to 65.6, *P*=.01) and, accordingly, less prolonged sitting time (−35.1 min/16 h, 95% CI −79.4 to 9.2, *P*=.12; which became significant after the removal of 2 influential [Cook’s *D* ≥.11] cases: −42.0 min/16 h, 95% CI −81.4 to −2.6, *P*=.04). Otherwise, there were only very small differences in sitting, standing, and stepping time changes. The size of the observed differences in work hour activities were all small (<the MDI) in addition to not reaching statistical significance.

### Aim 4: Acceptability and Perceptions of the LUMOback

At 3 months, questionnaire data were collected from 33 LUMOback users (13 male; aged 26-55 years; 4 team leaders, 1 senior leader, 15 managerial staff, and 13 general staff), and qualitative interviews were conducted with 22 LUMOback users (11 male; aged 26-57 years; 5 team leaders, 11 managerial staff, and 6 general staff) and 5 nonusers (3 male; aged 36-45 years; 1 team leader and 4 managerial staff). Several themes emerged from these 2 sources of data on LUMOback acceptability: (1) comfort of the LUMOback; (2) acceptability of the app, real-time feedback, and graphs; (3) use of the LUMOback features; (4) additional barriers; (5) perceived barriers in nonusers; (6) the perceived influence of others on LUMOback use; and (7) suggested improvements in an activity tracker. Supportive quotes are provided below and in Multimedia Appendix 3.

#### Comfort of the LUMOback

A majority of survey respondents (21/33, 64%) reported the LUMOback as only being “somewhat comfortable,” 9 participants (27%) reported the LUMOback to be “not at all comfortable,” and only 3 participants (9%) reported the LUMOback to be “comfortable” (see Multimedia Appendix 4). Comfort was identified as an issue in the telephone interviews. Reported issues included being conscious of wearing the LUMOback, the waist placement, sweating when wearing it, and, for women in particular, being hard to wear with clothing.

I think it was something that was hard to wear with some types of clothes. Not something that you could just put on and not think about. It was certainly thought about all the time while you were wearing it.

#3, female managerial staff, limited user

#### Acceptability of the App, Real-Time Feedback, and Graphs

Most users perceived the LUMOback and app to be “easy” to “extremely easy” to set up (n=22/33, 67%), use and navigate (25/33, 76%; see Multimedia Appendix 4), and the feedback graphs to be at least “somewhat useful” for creating awareness of sitting, standing, stepping, and good posture behaviors (27/33 (82%), 27/33 (82%), 26/33 (79%), and 26/33 (79%), respectively; see Multimedia Appendix 5). In the interviews, many participants reported that it was informative to get the real-time feedback from the LUMOback app:

I liked that you could track your results and see how well you are doing, I think that was the most benefit that you could constantly check where you're at.

#25, female general staff, infrequent user

#### Use of the LUMOback Features

Use of the sitting notification was modest, with half (16/33, 49%) not using it at all (Multimedia Appendix 5). Encouragingly though, most notification users (14/17, 82%) found the prompts to be at least “somewhat useful” for creating sitting awareness. Participants were most likely to select to be prompted for a sitting break every 30 min (11/17, 65%), compared with 15 min (2/17, 12%), 1 hour (3/17, 18%), or 2 hours (1/17, 6%).

The minimal use of the sitting notifications was echoed in the findings from the telephone interviews. When prompted, it appeared that not all participants were aware of the option to set sitting notifications or had forgotten. Out of those interviewees who did report using the sitting notification, some reported that the sitting notification was a “nice feature,” with one participant going on to say:

...[the sitting notification] actually became a useful conversation piece, you would be in a meeting and I would get a prompt and I’d stand up and I would explain to people what was going on, people thought it was interesting, so you are not just doing something weird like suddenly popping up in the middle of a meeting.

#21, male team leader, infrequent user

However, other users reported that the notifications were annoying and they turned them off, or they were easy to ignore.

The vibrating posture alerts were widely used and were highly discussed by participants. Some participants found the vibrating alerts beneficial:

...just the intrusiveness of it was actually quite good because it was always front of mind, you kind of couldn’t forget that it was there, especially when you had the pulse thing on.

#19, female managerial staff, limited user

However, many participants found that the vibrating alerts were a key barrier to wear as they were annoying and/or caused distraction in meetings. Although some participants were happy once disabling the vibrating alerts, others felt the LUMOback had lost its utility and purpose without the vibrations:

I did [turn the vibrating alerts off], but then I thought it’s a bit pointless because, not doing anything.

#17, male general staff, infrequent user

#### Additional Barriers

Some participants reported frustration having to repeatedly calibrate the LUMOback so that it was correctly measuring activity and posture and providing correct prompting:

You needed to recalibrate it every now and then and it would buzz as a result or it would be buzzing when you are in a right position and it was just a bit annoying.

#20, male managerial staff, frequent user

This was congruent with the questionnaire findings, with only just over half of participants (17/33, 52%) reporting the LUMOback was easy to calibrate (see Multimedia Appendix 4). Forgetting to wear the LUMOback was another barrier, with some participants reporting that the monitor used for assessments (ie, the activPAL) was easier to wear because “it just sat there all the time” and they did not need to remember to take it off and put it on each day (as it was taped to their thigh). Other barriers reported by a small number of participants included not wanting to wear the LUMOback at home because of the desire to sit and slouch and not be prompted during this time, having to charge the LUMOback, and the LUMOback app draining phone battery.

#### Perceived Barriers in Nonusers

Participants who did not wear the LUMOback were also interviewed and elaborated on their perceived barriers to uptake. A small number of participants reported set-up or syncing issues with the LUMOback. One of these participants mentioned that it would have been helpful having in-person assistance in setting up. A lack of interest, being too busy, or being forgetful also played a part for some participants not using the LUMOback.

#### The Perceived Influence of Others on LUMOback Use

Some participants reported that support from their team manager, the workplace champion, or other team members was related to uptake of the LUMOback. However, a majority of participants reported that their LUMOback use was not influenced by others because they did not talk about using the LUMOback or they could not see if other team members were wearing it.

#### Suggested Improvements in an Activity Tracker

Participants were also asked about their suggested improvements in activity trackers. The key desires for trackers included (1) being comfortable to wear, (2) being discreet, (3) being easy to use, (4) being waterproof, and (5) providing a range of accurate data.

Something I could just leave on that I wouldn't need to take on and off all the time, something that is waterproof, something that is maybe not so visible.

#3, female managerial staff, limited user

## Discussion

### Principal Findings

This is one of the first studies to comprehensively evaluate both the usage and acceptability of a wearable activity tracker when delivered as part of a workplace intervention. The real-time feedback was valuable to users, and greater use of the LUMOback appeared to assist with reducing the amount of daily prolonged sitting during the intervention. However, only 70% (43/61) of participants provided with a LUMOback used it. Among those who used the LUMOback, the amount of usage (left to the participant’s discretion) was very limited, with a median of 8 days of usage, and a maximum of 48 days of usage out of a possible 91 days. Being too busy, technology issues during setup, and a lack of interest were possible reasons for nonuse. Within users, comfort was a key reason for low usage of the LUMOback. Having lower self-perceived job control and weaker supervisor relationships were significantly associated with more LUMOback usage; while participants who were senior staff and team leaders, and participants with a better health profile also tended to use the LUMOback more than their counterparts.

Workplace tracker interventions have previously seen high levels of tracker uptake (96-99%) [13,27]. However, in line with the findings observed in this study (where uptake was 70%), uptake of trackers when offered in real-world workplace wellness programs can be more modest (25-100%) [9]. In this study, some nonusers reported a lack of interest in their LUMOback tracker. Tracker use is a personal decision, and some workers simply may not want or need a device to track their behavior. As such, offering trackers to everyone is not necessarily going to be a catch-all solution, but just one of many strategies a workplace could offer its workers. Issues with setup of the device may also have impeded its uptake. Although the participants in this study felt confident using their smartphone, work demands and technological difficulties may have meant that in-person set-up support was still needed. This finding, in line with a previous study reporting technology barriers to tracker uptake [28], suggests that investing in additional support at the initiation stage may be warranted. Indeed, in one study where such support was provided (eg, downloading the app and setting up the tracker for the participant), uptake was 100% and usage was high across the study (median use of 95% days across 16 weeks of intervention) [29].

Even among those who proceeded to use the belt-worn LUMOback, usage was low. Workplace studies evaluating trackers clipped on to the hip or shoe [12,13,27], worn on the wrist [14,30], or worn in a range of hip, wrist, and chest locations [31] have reported usage ranging from a mean use of 79% days across 6 weeks of intervention [12] to a mean use of approximately 69% days across 3 months of intervention [27], and 15% to upward of 89% of participants still using their tracker after 2 months [13,14,30,31]. All studies reporting high usage incorporated monetary incentives [13,27] or text message/email prompts [12] to encourage usage. Usage in the studies without these additional features [14,30,31] was typically lower (15-60% of participants still using their tracker after 2 months). These findings suggest that additional strategies may be needed to achieve high tracker usage.

Social support strategies, such as being part of a team, sharing results with others, and participating in workplace competitions, may also be associated with increased uptake and use of activity trackers [9,32]. In this study, senior and team leaders tended to use the LUMOback more than other employees. Participants in these roles likely had a higher responsibility for managing health and safety issues, had more direct contact with the workplace champion (who was at a similar managerial level), and as such may have been more motivated to use their LUMOback tracker. Senior and team leader support facilitated LUMOback uptake for some participants, but they did not discuss ongoing LUMOback use with their teams. A greater extent of role modeling and discussion might be useful to promote tracker usage and could provide a means to alleviate issues with setup and calibration.

Greater users of the tracker tended to be healthier, which is consistent with previous research [15,33]. A novel finding was that participants with high self-perceived job control and supervisor relationships used the LUMOback for significantly fewer days. It may have been that these participants felt more freedom and confidence in their work roles to discontinue use of the tracker. Participants who had musculoskeletal problems at baseline used the LUMOback for fewer days than those without problems. It is possible that minor back pain following the posture advice, which was reported by 3 participants in the adverse outcomes of the trial [18], was a reason for less use in those participants who already had musculoskeletal problems at baseline. The waist-worn belt may have also been particularly troublesome for those with pre-existing back problems.

Comfort of the LUMOback appeared to be a key contributor to the low overall usage identified in this study. Participants reported dislike of the belt placement, and some noted that the LUMOback was difficult to wear with clothing. Similar findings have been reported with the Actigraph GT3X+ when attached via a belt [34] and for trackers worn tightly secured to the body [31]. Alternatives to belts, such as wristbands and clips, may be especially useful for long-term use with office clothing. However, the wrist and hip wear positions suited to wristbands and clips are not ideal for measuring sitting time [20] as they can lead to standing being misidentified as sitting. Other barriers identified by participants in this study and across other tracker research include a lack of perceived data accuracy, forgetting to use or charge the tracker, and difficulty using the tracker [14,30,31]. These findings suggest that, even with additional support, trackers need to be comfortable, accurate, discreet, and easy to use to facilitate ongoing use.

Consistent with other workplace tracker studies [14,31], we saw very limited usage of the LUMOback after 3 months (ie, only 1 out of the 21 participants whom we could follow up). However, the need for continuous self-monitoring of behavior is debatable [35]. Disuse of a tracker after a short period could indicate that participants have learned about their behavior and made changes rather than indicating failure to change [31]. Alternatively, the intermittent usage observed for some participants may reflect that self-monitoring provides a way to reidentify and adjust behavior as needed. Our findings did not suggest that the behavioral impact of the LUMOback is limited to the time frame over which it was worn; usage during the activity monitoring did not appear to predict behavior change any better than usage over the intervention generally, much of which occurred early on. A period of initial tracking may be sufficient to facilitate 3-month behavior changes. However, further research is needed to understand the interplay between tracker use and behavior.

Encouragingly, even within a group who used the LUMOback to a limited degree, larger favorable behavior changes during the intervention were seen in those with high usage compared with those with low or no usage. Greater usage during the intervention was significantly associated with overall time per day spent in prolonged sitting and, although there should be caution in interpreting this result, time spent in nonprolonged sitting during work hours. Unlike the majority of commercially available activity trackers [20], the LUMOback used posture measurement to detect and prompt on sitting time, which may be why this particular tracker was able to facilitate improvements in prolonged sitting time during the intervention. However, the LUMOback is no longer commercially available. Thus, it is recommended that current and future commercial activity trackers include features to distinguish between sitting and upright postures so that they can appropriately measure and prompt on sitting time.

Notably, use of the LUMOback was not significantly associated with changes in sitting, standing, and stepping time. Greater changes in these behaviors may require greater use of the LUMOback and/or additional behavioral strategies. Environmental modifications to the workplace (eg, sit-stand workstations) and/or changes to the design of work tasks (eg, activity-based working, where workers can change workspace depending on work task) may also be needed to support regular shifts between sitting, standing, and moving.

### Strengths and Limitations

A key study strength was that use of the LUMOback was self-directed by the participant and was provided as part of a broader workplace program. Thus, the findings are likely to be indicative of how a tracker may be implemented and used as part of a real-world worksite-driven program. The evaluation over the longer term enabled the examination of how the LUMOback was being used over time (and by whom), whereas the qualitative investigation added depth to the findings by exploring the reasons for lack of uptake and ceased use. It is recommended that future tracker evaluations take this mixed-methods approach.

This study was not powered a priori on the aims of this paper. Confidence intervals around estimates indicated that substantial differences in sociodemographic and health- and job-related predictors of usage and substantial differences in activity changes by usage may have been missed due to inadequate precision. Moreover, the limited differences in activity changes by usage may in part be due to a mismatch between when participants used their LUMOback and when we captured their behavior. Evaluating usage and behavior over a more similar time frame (the assessment period vs the intervention) did not tend to strengthen associations; however, our examination would not detect very short-term effects of usage on behavior (eg, on the same day or at the same time). This may be an important question to address in a sample with sufficient usage. Biases may have had an impact on the results. Due to the small sample size, it was not possible to adjust for all potential confounders. Information was not collected on those who declined participation or who were never approached. LUMOback nonusers were also more likely to drop out of the study than LUMOback users. This attrition bias suggests our results, if anything, are likely to underestimate the associations of usage with behavior change. Although only some participants participated in the telephone interviews, interviews were conducted in both LUMOback users and nonusers, and a range of perspectives was collected. Generalizability is limited to the type of tracker used (belt-worn tracker focused on posture and sitting) and to the context of its use. Notably, it was allocated and provided by others (not chosen or purchased by the user) in the context of a team-based workplace-delivered organizational intervention. Use of a tracker that is self-selected by participants might lead to greater uptake and/or longer usage. Self-selection may especially encourage usage in those with high job control who may be accustomed to having a high level of autonomy in their working life.

### Conclusions

A tracker that provides real-time feedback on sitting time was associated with the reduction of prolonged sitting time during a workplace-delivered sitting-reduction intervention. The tracker evaluated was not particularly suitable for ongoing use in office workers, but this study has provided insight into barriers and facilitators for uptake and ongoing tracker use in this population.

## Appendix

Multimedia Appendix 1 Characteristics of participants randomized to the ORG+Tracker intervention overall and by usage group.

Multimedia Appendix 2 Participant flow diagram.

Multimedia Appendix 3 Additional participant quotes.

Multimedia Appendix 4 Acceptability of the LUMOback.

Multimedia Appendix 5 Use and perceived usefulness of the LUMOback features.

## Abbreviations

MDI: minimum difference of interest
