# Supplementary material for: Usage, Acceptability, and Effectiveness of an Activity Tracker in a Randomized Trial of a Workplace Sitting Intervention: Mixed-Methods Evaluation
Source: Interact J Med Res. 2018 Mar 2;7(1):e5. doi: 10.2196/ijmr.9001 (PMC5856932; doi:10.2196/ijmr.9001)
Supplement: Multimedia Appendix 1 [file ijmr_v7i1e5_app1.pdf]

Multimedia Appendix 1. Characteristics of participants randomized to the ORG+Tracker intervention overall and by usage group.

	Group ORG+Tracker	Frequent users	Infrequent users	Limited users	Non-users
	n=66 [a]	n=9 [a]	n=16 [a]	n=13 [a]	n=24 [a]
Female	53% (35)	56% (5)	56% (9)	46% (6)	54% (13)
Age, years	37.6±7.8	41.0±8.5	36.1±7.7	39.2±8.5	36.9±7.2
Aged ≥36 years	52% (33)	67% (6)	38% (6)	54% (7)	57% (12)
BMI, kg/m^2	24.1±3.4	23.9±3.2	23.4±2.6	27.0±4.3	23.6±2.9
Normal weight	74% (37)	67% (4)	93% (14)	40% (4)	76% (13)
Overweight	16% (8)	33% (2)	0% (0)	30% (3)	18% (3)
Obese	10% (5)	0% (0)	7% (1)	6% (1)	
			30% (3)		
Current smoker	9% (5)	0% (0)	7% (1)	8% (1)	16% (3)
University education	86% (54)	78% (7)	75% (12)	92% (12)	90% (19)
Preferred time spent sitting at work ≥50%	31% (18)	33% (3)	33% (5)	25% (3)	32% (6)
Knowledge of sitting & health [b]	3.9±0.5	3.8±0.4	4.0±0.4	3.9±0.5	3.8±0.5
Smartphone confidence [b]	3.9±0.9	3.7±0.9	3.9±0.9	4.0±1.0	3.9±0.9
Previous activity app or device use	34% (19)	44% (4)	27% (4)	42% (5)	29% (5)
**Health variables**					
Musculoskeletal [c]					
Upper body problems	64% (37)	56% (5)	73% (11)	83% (10)	53% (10)
Back problems	59% (34)	56% (5)	60% (9)	58% (11)	67% (8)
Lower extremity problems	26% (15)	11% (1)	27% (4)	42% (5)	21% (4)
Stress [d]	6.2±2.5	4.9±1.9	6.1±2.5	6.0±2.3	7.1±2.9
Physical health quality of life [e]	51.8±6.8	53.9±7.2	50.7±7.0	49.1±7.6	52.9±6.1
Mental health quality of life [e]	46.1±10.3	52.9±4.0	46.2±10.8	40.8±13.4	46.2±7.4
**Work variables**					
1.0 Full-time equivalent	92% (59)	100% (9)	88% (14)	92% (12)	91% (20)
Job category					
Team leader	9% (6)	11% (1)	19% (3)	0% (0)	4% (1)
Senior leader	3% (2)	11% (1)	0% (0)	0% (0)	4% (1)
Managerial staff	58% (38)	44% (4)	31% (5)	77% (10)	67% (16)
Other general staff	30% (20)	33% (3)	50% (8)	23% (3)	25% (6)
Job performance [d]	7.7±0.9	7.8±0.5	7.5±1.0	7.5±1.0	8.1±0.7
Job control [d]	7.0±1.9	6.8±1.0	6.1±2.5	6.7±1.5	7.6±1.5
Work satisfaction [d]	6.6±1.6	6.8±0.8	6.1±1.9	6.2±1.9	7.0±1.3
Supervisor relations [d]	6.8±1.8	6.4±1.1	6.4±2.3	6.5±1.9	7.5±1.5
Weekday work hours/day [f]	9.6±1.0	9.3±0.6	9.4±1.2	9.4±0.9	9.9±1.0
**Activity variables**					
Work hours (min/10h workday)					
Sitting	453.0±55.9	452.2±37.4	464.8±40.4	478.9±39.0	431.5±73.7
Prolonged sitting	254.8±89.8	210.9±72.1	255±111.8	312.4±63.2	239.4±72.6
Standing	100.2±50.8	106.4±30.9	89.6±31.4	79.7±29.0	115.0±73.6
Stepping	46.8±15.1	41.3±14.5	45.6±14.6	41.4±14.6	53.5±14.5

Overall hours (min/16h day)					
Sitting	627.0±65.2	604.0±62.3	643.5±75.4	654.2±49.7	611.9±62.5
Prolonged sitting	333.3±96.9	267.5±77.0	345.1±130.6	378.8±60.7	322.9±76.2
Standing	216.9±56.3	242.0±46.4	201.7±61.5	194.3±37.9	225.6±61.2
Stepping	116.1±26.3	114.0±28.1	114.7±26.6	111.5±20.7	122.5±28.8

Data are % (n) or mean±SD as measured at baseline.

[a] Total n as shown but table excludes (listwise) participants with missing data (frequent-/infrequent-/limited-/non users/unknown usage): sex, job category (0/0/0/0/0); full-time status (0/0/0/2/0); activity data (0/0/1/1/0); work activity data (0/0/1/3/0); weekday work hours, age, education (0/0/0/3/0); musculoskeletal items, smoking status, preferred time spent sitting, knowledge of sitting and health (0/1/1/5/1); quality of life, stress, job performance, supervisor relations, work satisfaction, previous app/device use (0/1/1/7/1); job control (0/1/1/8/1); smart phone confidence (0/1/2/8/1); and, BMI (3/1/3/7/2).
Totals for each of the usage categories will not always add to the ORG+Tracker group total as there were four participants with missing usage data.
[b] per 1 point, 1 to 5 scale, higher numbers indicate more knowledge of the health impacts of sitting, or greater smartphone confidence
[c] The 36-item version of the Nordic Musculoskeletal Questionnaire, [23] modified to measure problems in the last month. Issues identified as causing trouble were collapsed into categories of upper body problems (i.e., neck, shoulders, elbows, and wrists/hands), back problems (i.e., upper back, lower back, and hips/thighs/buttocks) or lower extremity problems (i.e., knees, and ankles/feet).
[d] 1 to 10 scale, higher numbers indicate more favourable workplace scores, or greater stress levels
[e] 0 to 100 scale, higher numbers indicate better quality of life
[f] Average weekday work hours were calculated from baseline work diaries.
