# Supplementary figures and images for: Usage, Acceptability, and Effectiveness of an Activity Tracker in a Randomized Trial of a Workplace Sitting Intervention: Mixed-Methods Evaluation
Source: Interact J Med Res. 2018 Mar 2;7(1):e5. doi: 10.2196/ijmr.9001 (PMC5856932; doi:10.2196/ijmr.9001)

Multimedia Appendix 2. Participant flow diagram. 3M = 3 months, 12M = 12 months.

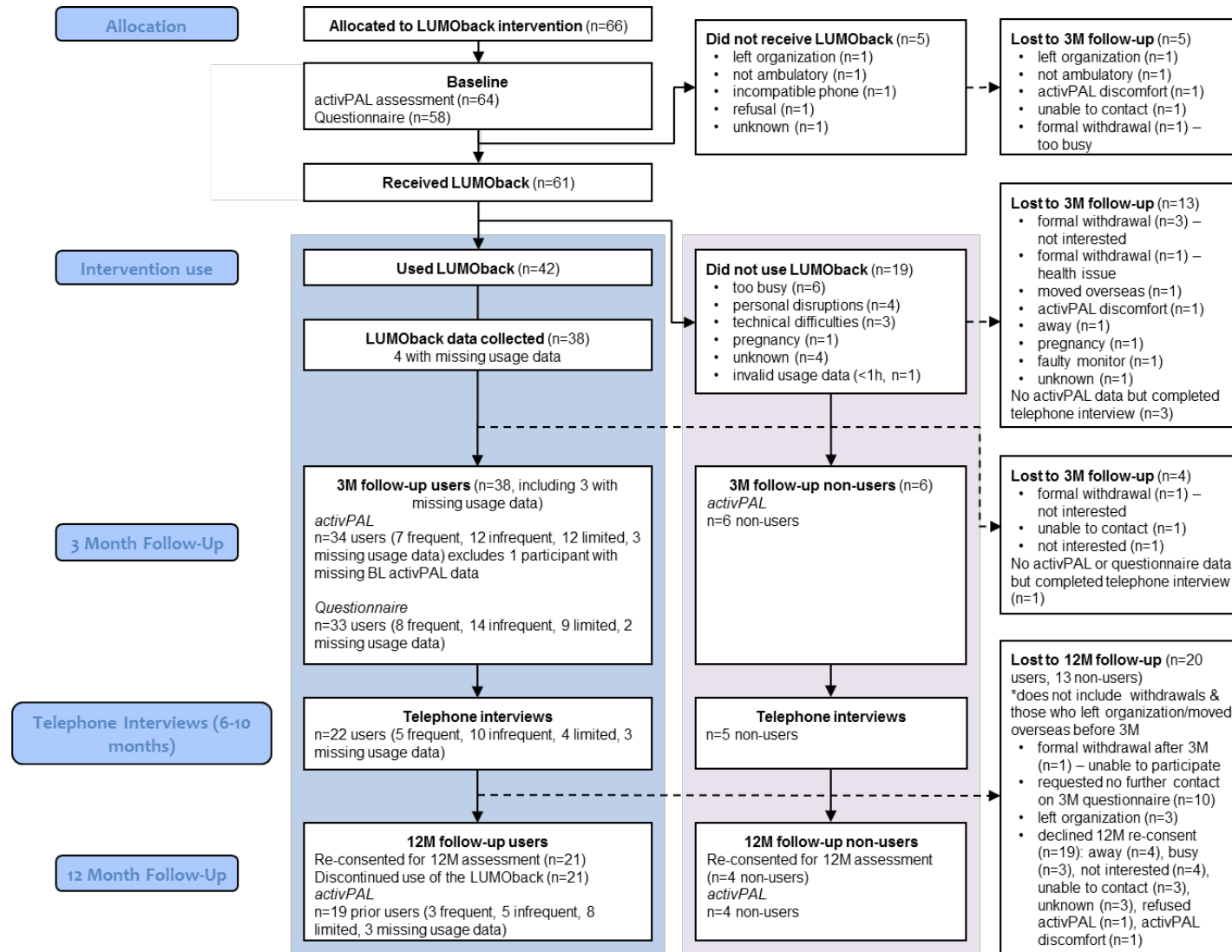

Supplement: Multimedia Appendix 2 [file ijmr_v7i1e5_app2.pdf]
