# Supplementary material for: Usage, Acceptability, and Effectiveness of an Activity Tracker in a Randomized Trial of a Workplace Sitting Intervention: Mixed-Methods Evaluation
Source: Interact J Med Res. 2018 Mar 2;7(1):e5. doi: 10.2196/ijmr.9001 (PMC5856932; doi:10.2196/ijmr.9001)
Supplement: Multimedia Appendix 3 [file ijmr_v7i1e5_app3.pdf]

Multimedia Appendix 3. Additional participant quotes.

Themes	Quote/s
Comfort of the LUMOback	*"just in general I found it really uncomfortable going around my waist, it didn't fit well with some of my clothing yeah just that was the main barrier for me"* (#5, female managerial staff, infrequent user)
Acceptability of the app, real-time feedback and graphs	*"I'll sort of check it and see how many hours I have been sitting down and go oops that's a bit too much and if I had just looked at it and been oh I have been spending a lot of time sitting down today I will make more of an effort the next day to move around more anyway, and I think just having that facility close by where you can check on your progress during the day that was a really good thing to have."* (#27, female general staff, frequent user)
Use of the LUMOback features – sitting notifications	*"I sort of really forgot that it had that, I probably should have actually because I really do sit down too much."* (#27, female general staff, frequent user)<br><br>*"[the sitting notification is] actually quite a nice feature that, every now and then you get a reminder to stand up"* (#17, male general staff, infrequent user)
Use of the LUMOback features – vibrating alerts	*"I think it's great to have them on sort of a more disciplined setting when you are sitting at your desk but I found it a bit distracting in meetings etc"* (#8, female managerial staff, frequent user)
Additional barriers	*"I kept kind of forgetting to put it on in the mornings whereas the thing on my leg it just sat there all the time so I didn't have to remember to put it on or take it off it just sat there"*(#25, female general staff, infrequent user)
Perceived barriers in non-users	*"I tried it a couple of times at home and it just wouldn't work and then I just gave up on it….would have been helpful if there was someone who really knew how to operate these things that he could spend 5 minutes with getting it set up."* (#22, male managerial

	staff, non-user)<br><br>*"I was too busy I guess that is a reason but also I didn't want to, I probably just didn't want to, I didn't feel like from a personal point of view that I really wanted to wear that around"* (#12, male managerial staff, non-user)
The perceived influence of others on LUMOback use	*"I remember people would just go oh yeah I am wearing that but then you know sort of forgot about it."* (#21, male team leader, infrequent user)<br><br>*"we didn't really talk about it much as a group it was sort of like incidental sort of conversation"* (#27, female general staff, frequent user)
Suggested improvements in an activity tracker	*"for me it is comfort and something that I don't really have to think about because it's just there"* (#25, female general staff, infrequent user)
