# Supplementary material for: Usage, Acceptability, and Effectiveness of an Activity Tracker in a Randomized Trial of a Workplace Sitting Intervention: Mixed-Methods Evaluation
Source: Interact J Med Res. 2018 Mar 2;7(1):e5. doi: 10.2196/ijmr.9001 (PMC5856932; doi:10.2196/ijmr.9001)
Supplement: Multimedia Appendix 4 [file ijmr_v7i1e5_app4.pdf]

Multimedia Appendix 4. Acceptability of the LUMOback, n=33

	Extremely comfortable /easy, % (n)	Comfortable / easy, % (n)	Somewhat comfortable /easy, % (n)	Not at all comfortable /easy, % (n)	Not sure, % (n)
Comfortable	0% (0)	9% (3)	64% (21)	27% (9)	0% (0)
Ease of:					
Set-up	9% (3)	58% (19)	24% (8)	6% (2)	3% (1)
Navigation & use	6% (2)	70% (23)	21% (7)	3% (1)	0% (0)
Calibration	3% (1)	49% (16)	49% (16)	0% (0)	0% (0)

Percentages do not add to 100% due to rounding.
