# Supplementary material for: Usage, Acceptability, and Effectiveness of an Activity Tracker in a Randomized Trial of a Workplace Sitting Intervention: Mixed-Methods Evaluation
Source: Interact J Med Res. 2018 Mar 2;7(1):e5. doi: 10.2196/ijmr.9001 (PMC5856932; doi:10.2196/ijmr.9001)
Supplement: Multimedia Appendix 5 [file ijmr_v7i1e5_app5.pdf]

Multimedia Appendix 5. Use and perceived usefulness of the LUMOback features, n=33

	Extremely useful, % (n)	Useful, % (n)	Somewhat useful, % (n)	Not at all useful, % (n)	Not sure, % (n)	Did not use, % (n)
**Feedback graphs**						
Sitting awareness	12% (4)	39% (13)	30% (10)	6% (2)	6% (2)	6% (2)
Standing awareness	9% (3)	42% (14)	30% (10)	6% (2)	6% (2)	6% (2)
Step count awareness	15% (5)	30% (10)	33% (11)	6% (2)	9% (3)	6% (2)
Posture awareness	18% (6)	30% (10)	30% (10)	9% (3)	6% (2)	6% (2)
**Sitting notifications**						
Sitting awareness	3% (1)	21% (7)	18% (6)	3% (1)	6% (2)	49% (16)
**Vibrating alerts**						
Posture awareness	12% (4)	33% (11)	24% (8)	12% (4)	3% (1)	15% (5)

Percentages do not add to 100% due to rounding.
